# Interplay of Muscle Architecture, Morphology, and Quality in Influencing Human Sprint Cycling Performance: A Systematic Review

**DOI:** 10.1186/s40798-024-00752-2

**Published:** 2024-07-19

**Authors:** Saul Martin-Rodriguez, Juan J. Gonzalez-Henriquez, Iker J. Bautista, Jose A. L. Calbet, Joaquin Sanchis-Moysi

**Affiliations:** 1 Department of Physical Education, University of Las Palmas de Gran Canaria, Las Palmas de Gran Canaria, 35017 Spain; 2 Research Institute of Biomedical and Health Sciences (IUIBS), Canary Islands, Las Palmas de Gran Canaria, 35017 Spain; 3 Department of Mathematics, University of Las Palmas de Gran Canaria, Las Palmas de Gran Canaria, Spain; 4 Institute of Sport, Nursing, and Allied Health, University of Chichester, Chichister, PO19 6PE UK; 5 Department of Physical Performance, The Norwegian School of Sport Sciences, Postboks, 4014 Ulleval Stadion, Oslo, 0806 Norway

**Keywords:** Muscle volume, Cross-sectional area, Pennation angle, Fascicle length, Muscle Thickness

## Abstract

**Background:**

This systematic review aimed to discern the relationships between muscle morphology, architecture, and quality with sprint cycling performance while considering the multifaceted nature of these relationships across diverse studies.

**Methods:**

Employing the PRISMA guidelines, an exhaustive search was performed across four primary databases: MEDLINE/PubMed, Web of Science, CINAHL Complete, and SPORTDiscus. The Methodological Index For Non-Randomised Studies (MINORS) was used to assess the methodological quality of the included studies. Out of 3971 initially identified records, only 10 studies met the eligibility criteria.

**Results:**

These investigations underscored the robust relationship of quadriceps muscle volume with peak power output (R^2^ from 0.65 to 0.82), suggesting its pivotal role in force production. In muscle architecture, the pennation angle and fascicle length showed varied associations with performance. Furthermore, muscle quality, as denoted by echo intensity, showed preliminary evidence of a potential inverse relationship with performance. The methodological quality assessment revealed varied scores, with the most consistent reporting on the aim, endpoints, and inclusion of consecutive patients. However, limitations were observed in the prospective calculation of study size and unbiased assessment of study endpoints.

**Conclusion:**

Our findings indicate that muscle volume is a major determinant of sprint cycling performance. Muscle architecture and quality also impact performance, although in a more intricate way. The review calls for standardised methodologies in future research for a more comprehensive understanding and comparability of results.

**PROSPERO registration number:**

CRD42023432824 (https://www.crd.york.ac.uk/PROSPERO/display_record.php?RecordID=432824).

**Supplementary Information:**

The online version contains supplementary material available at 10.1186/s40798-024-00752-2.

## Key Points

• Muscle size matters: greater muscle volume and cross-sectional area correlate strongly with peak power output, emphasising the importance of muscle size in sprint cycling.

• Pennation angle positively correlates with peak power output, but varied associations indicate the existence of other influencing factors, highlighting the complexity of muscle architecture in sprint performance.

• Preliminary evidence suggests lower echo intensity, indicating higher muscle quality, may be linked to better sprint performance, urging further exploration of this relationship in athletes.

**Supplementary Information:**

The online version contains supplementary material available at 10.1186/s40798-024-00752-2.

## Background

Sprint cycling is a high-intensity, explosive discipline, where the ability to generate maximal power is crucial for success [1]. Bicycling requires the coordinated extension and flexion of multiple joints, notably encompassing the hip, knee, and ankle [2]. By combining electromyography and force measurements during pedalling it has been shown that the knee extensors, hip extensors, ankle plantar flexors, knee flexors, and hip flexors muscles contribute 39, 27, 20, 10, and 4%, respectively, to the power generated during cycling [3]. Monoarticular thigh muscles - comprising the gluteus maximus, vastus lateralis, vastus medialis, tibialis anterior, and soleus - are predominantly responsible for generating the forces transmitted to the pedals. Of these, the vastus medialis and vastus lateralis manifest peak activity commencing from the top dead centre (0º) of the pedal cycle and continuing halfway (90º) through the propulsion phase (0-180º) [4–6]. Conversely, biarticular muscles, including the biceps femoris, semitendinosus, semimembranosus, rectus femoris, and the medial and lateral heads of the gastrocnemius, primarily exert control over the direction of the forces applied to the pedals [6–8]. Thus, the muscles of the lower extremities are submitted to specific mechanical demands during cycling and some morphological and architectural features are likely more suitable for maximising sprint performance. However, little is known about the morphological and architectural determinants of peak power output during sprint cycling [9].

Muscle morphology (e.g., muscle volume or mass) and architecture (e.g., the geometric arrangement of muscle fibres) are crucial for force production in cycling [9, 10]. Muscle volume refers to the total amount of muscle tissue within a specific anatomical region, and it is typically calculated by integrating cross-sectional area (CSA) measurements along the length of the muscle. Muscle volume and anatomical CSA are measurements of muscle size often determined by magnetic resonance or ultrasound imaging that present a strong relationship (*r* > 0.73) with muscle strength [11, 12]. Functional magnetic resonance imaging (MRI) indicates that the quadriceps muscle is the main contributor to power generation [13], however, peak power during sprinting cycling can also be predicted by the sum of the lean mass of the lower extremities [14]. Muscle architectural features include muscle thickness (MT), pennation angle (PA), and fascicle length (FL) [15]. Muscles with longer fibres and greater pennation angles can typically produce more power [16]. This is because longer fibres have more sarcomeres in series, allowing faster contraction speeds, while a higher pennation angle reflects a higher number of muscle fibres and/or increased cross-sectional area of the individual fibres (thus more sarcomeres) in parallel with an enhanced capacity for force generation [17]. More recently, indirect indices of muscle quality, like echo intensity, have been proposed to assess the composition of the muscle which may influence its performance potential. Echo intensity is a B-mode ultrasound-derived measure related to the strength of the returned signal after sending an ultrasonic wave through tissue. Several factors may influence echo intensity values and interpretation, such as methodological factors (i.e., subcutaneous fat correction and probe tilt), previous exercise (muscle damage), hydration, water distribution between intra and extracellular compartments, age, sex, ethnicity, adiposity, muscle size, strength, and connective tissue, among others [ [18, 19]. Additionally, muscle temperature and fascicle angle have been shown to be negatively associated with echo intensity [20]. These findings underscore the importance of considering both physiological and methodological factors when interpreting echo intensity measurements. Low echo intensity has been related to superior muscle quality [21], whereas high echo intensity has been associated with muscle impairment and disease [22]. Some authors have observed an inverse relationship between echo intensity and cardiorespiratory performance in aged persons, suggesting that connective and adipose tissue accumulation may be detrimental to cardiorespiratory capacity [23]. Echo intensity is negatively associated with functional capacity in the elderly [24–27] and lower muscle power [26, 28, 29].

While some studies have explored the impact of muscle characteristics on athletic performance [30–34], a comprehensive synthesis of the literature focusing specifically on sprint cycling performance is lacking. This systematic review offers an opportunity to critically analyse the existing body of evidence, identify trends, and assess the overall strength of associations between muscle morphology, muscle architecture, and muscle quality with sprint cycling performance. This systematic review aimed to determine the extent to which muscle morphology, muscle architecture, and muscle quality are associated with sprint cycling performance. By synthesising findings from various studies, we seek to provide a comprehensive overview of the current state of knowledge and identify potential gaps and limitations in the existing research.

## Methods

### Study Design

The design of this systematic review was developed through the Reporting Items for Systematic Reviews and Meta-analysis (PRISMA) statement guidelines [35]. The protocol was pre-registered on PROSPERO (CRD42023432824) before searches and data extraction. Before the registration, a detailed search was performed on PROSPERO (https://www.crd.york.ac.uk/PROSPERO/) to identify similar reviews. The PRISMA checklist is available in Supplementary File1.

### Search Strategy

The primary search focused on studies reporting on the associations of muscle architecture features with sprint cycling performance. An electronic database search for the articles published online or in print up to July 2023 was performed in four databases: MEDLINE/PubMed, Web of Science, CINAHL Complete, and SPORTDiscus (via EBSCOhost). The Cochrane database was also searched for a potentially similar review. To reduce publication bias, the search was performed with no restrictions on date or language. Search strings for electronic databases are shown in Supplementary File2.

The Participants, Interventions, Comparators, Outcomes, and Study Design (PICOS) framework [36] was used to build search criteria for electronic databases. The PICOS consists of terms related to sprint performance, muscle morphology, architecture, and diagnostic imaging techniques. The search strings used for the other databases were adapted using the Polyglot Search Translator Tool (https://sr-accelerator.com/#/polyglot) [37]. These search strings are reported in Supplementary File1. An automatic online deduplicator tool (https://sr-accelerator.com/#/deduplicator) was employed to identify and remove duplicate publications. After removing duplicates, the resulting non-duplicated references were uploaded to a reference management tool (EndNote 20, Clarivate Analytics, PA, USA) to manually search for two reasons: (1) to manually eliminate possible duplicates that the online tool did not identify because they were in another language, and (2) to identify and manage the articles of interest by title and abstract. From the initial search, the titles and abstract were reviewed to exclude any irrelevant study. The full texts of the remaining studies were then retrieved and read independently by two authors (SMR and IJB) to determine whether the studies met the inclusion criteria. Any disagreement was resolved by consensus with a third author (JJGH).

### Eligibility Criteria

The main selection of studies was performed by applying the following inclusion criteria: a) articles must be strictly focused on investigating associations, i.e., using the Pearson product-moment correlation coefficient or Spearman´s rank correlation coefficient, between muscle morphology, architecture, or quality and sprint cycling performance, (b) articles must contain at least one measurement of muscle architecture and one measurement of sprint cycling performance, (c) articles must evaluate muscle morphology, architecture, or quality with the following diagnostic imaging devices: MRI or ultrasound imaging. Grey literature was excluded, i.e., studies were required to be published in a peer-reviewed journal indexed in the Journal of Citations Reports or the SCImago Journal & Country Rank. Conference abstracts were also excluded due to the difficulty in obtaining full methods and complete data sets. Finally, studies were excluded if they included individuals with known pathologies and/or injuries. Due to the limited number of studies, we decided not to restrict by sex. In the case of studies measuring muscle architecture in several regions of the muscle of interest, only the assessments obtained in the mid portions, i.e., 50% of the length, were retained for data analysis unless most articles incorporate evaluations in multiple sites of the muscles of interest. A complete list of reports excluded for eligibility is displayed in Supplementary File3.

### Data Extraction

To identify and extract representative data from all the included articles, publications were analysed by one author (SMR) and cross-checked by a second (IJB). The following data were extracted and coded for authors in an Excel spreadsheet: year of publication, research design (i.e., observational study), sample size, sex, age, body mass, the Pearson Product Moment Correlation (r) or Spearman´s rank correlation coefficient (rho) of muscle morphology (i.e., volume), muscle architecture features (i.e., PA, FL, MT, echo intensity, volume, CSA), and the devices used to assess sprint performance and muscle morphology, architecture, and quality. Where data extracted were not available from tables, figures, or the results section, authors were contacted a maximum of three times over 4 weeks to request the information and allow a final decision on inclusion to be made. Data extraction was completed in duplicate independently by two of the co-authors (SMR and IJB). A third co-author checked the similarity between the data extracted by these two co-authors (JJGH). Any discrepancies were reviewed and agreed upon by all assessors after discussion. This method was tested on the first five studies chosen at random before commencing data extraction. The data were extracted in the form of percentages, ranges, correlations, and regressions, aligning with the outcomes reported by the studies analysed. Pearson´s and Spearman´s rho correlations were interpreted as reported elsewhere [38, 39].

### Assessment of Methodological Quality

Two researchers (SMR and IJB) independently assessed the methodological quality of the studies using the Methodological Index For Non-Randomized Studies (MINORS) [40]. This scale consists of 12 items grouped into three sections: study design, analysis, and outcomes. Each item is scored on a scale of 0 to 2, with a higher score indicating better methodological quality. The global ideal score is 16 for non-comparative studies and 24 for comparative studies. A third evaluator (JJGH) made the final decision in cases of disagreement between the scores provided. Finally, the risk of bias was not assessed due to the lack of validated tools to assess the risk of bias in the type of studies included in this review.

## Results

### Characteristics of Included Studies

**Fig. 1:**
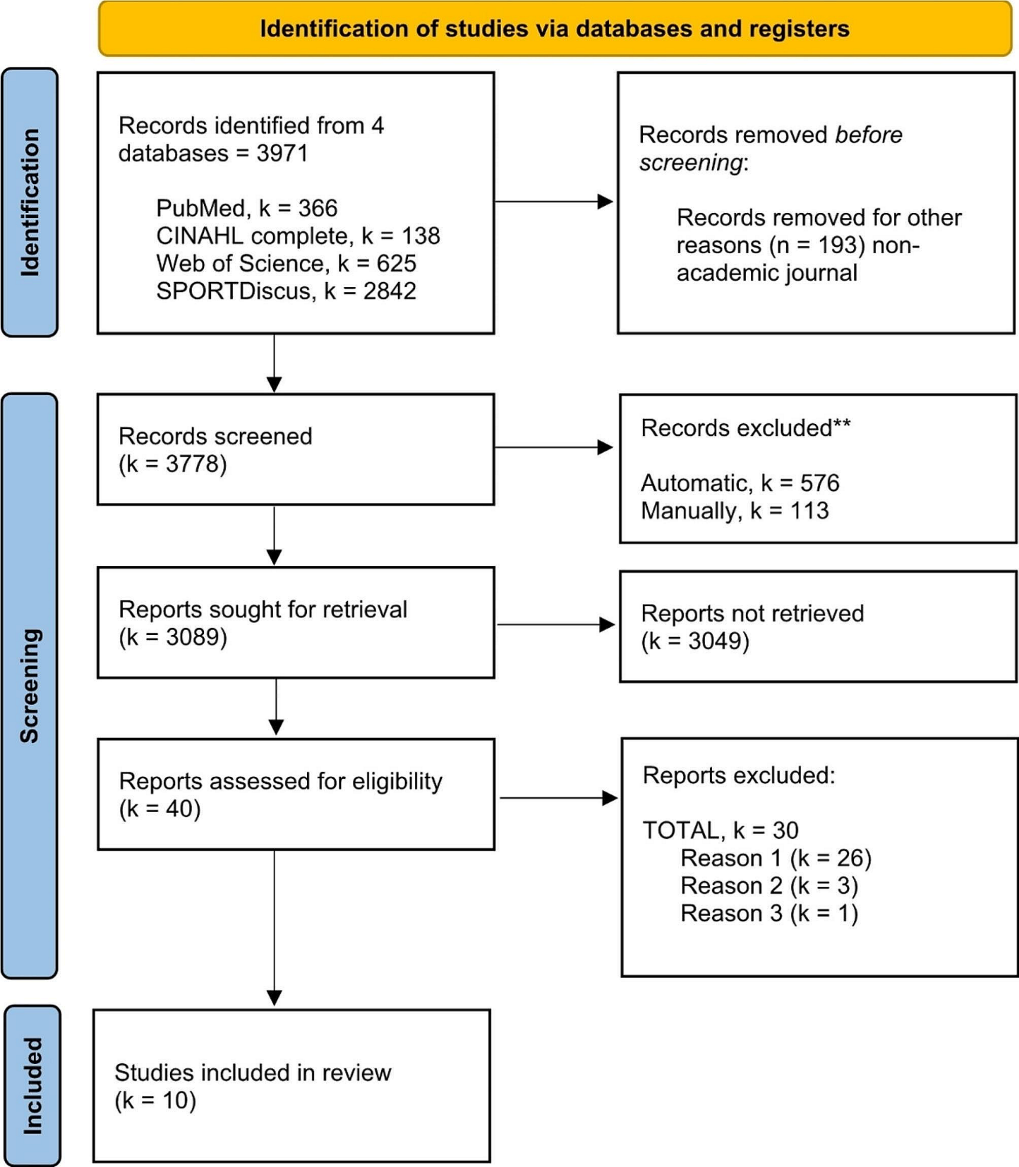
PRISMA flow chart. The reasons for excluding reports (bottom right-hand box) are detailed below. Reason 1: Studies not investigating associations, i.e., using the Pearson product-moment correlation coefficient or Spearman´s rank correlation coefficient, between muscle architecture, morphology, or quality, and cycling sprint performance; Reason 2: Articles not evaluating muscle morphology, architecture, and quality with the following diagnostic imaging devices: computed tomography, magnetic resonance imaging, or ultrasound; Reason 3: Review articles

The literature search resulted in 3971 records identified through four electronic databases. Before the screening, 193 records were removed from SPORTDiscus under the criterion of non-academic journals. A total of 3778 records were screened, and 689 were excluded due to duplication. From the remaining 3089, just 40 articles accomplished the eligibility criteria. Of these, 30 were excluded for different reasons (see Supplementary File3). Finally, 10 studies analysing the relationship between muscle morphology, architecture, or quality with sprint cycling performance were included in the qualitative synthesis of this systematic review. All studies included in this systematic review were cross-sectional observational studies published between 1997 and 2022. The flowchart is shown in Fig. 1.

### Participants

The total sample was composed of 254 participants [222 males (85.4%) and 32 females (15.6%)]. Sample sizes ranged between 10 [41] and 58 [42]. The ages of the subjects ranged between 9 [43] and 33 years [44]. Three articles included elite athletes [45–47], while six included cyclists [44, 45, 47–50]. Only one study evaluated the maximum oxygen consumption [45].

### Diagnostic Imaging and Sprint Performance Devices

All the studies employed ultrasound imaging devices except one [43]. Two studies used 3D ultrasound imaging [45, 46], while the rest employed 2D technology. Two articles included MRI devices of 0.5 and 1.5 T, respectively [43, 47].

Half of the studies (50%) used the Monark 894 E Peak Bike [44–46, 49] or the 814 E [43]. The rest employed the Lode Excalibur [41, 42], the Watttbike [48, 50], or the Schoberer ergometer [47].

### Muscle Morphology, Architecture, and Muscle Quality Variables

All articles reported the evaluation of lower extremities, while only one article also reported muscles from the upper extremities [48]. All the studies analysed the dominant leg, whereas just one study analysed the non-dominant leg [41]. None of the included studies reported the criteria to establish leg dominance. The most reported muscles were the rectus femoris and the vastus lateralis (Table 1). All the included articles analysed muscle architecture at only one site (i.e., 50% of the vastus lateralis), except for two that included several measurement sites [42, 50].

**Table 1:** Study characteristics

Reference	Study design	*N* (M/F)	Population (age (years))	Dominant/Non-Dominant leg measures	Muscle analysed	Muscle morphology outcomes	Muscle architecture outcomes	Fascicle length US calculation method	Type of sprint	Sprint performance outcomes
Welsman et al. [43]	CR	32 (16/16)	9–10	Yes/No	Right thigh	Volume	N.A.	N.A.	Wingate 30 s	1-s peak power, 30 s mean power
McCormack et al. [41]	CR	10 (0/10)	19.5 ± 1	Yes/Yes	VL	N.A.	MT, PA	N.A.	Wingate 30 s	Peak power during the Wingate, 30 s mean power
Lee et al. [48]	CR	12 (12/0)	21 ± 0.9	Yes/No	RF, VL, rectus abdominis, erector spinae	N.A.	MT	N.A.	Wingate 30 s	5-s peak power, 30 s mean power
van der Zwaard et al. [46]	CR	18 (12/6)	27 ± 2	Yes/No	VL	Volume	pCSA, FL, PA	Not reported	Wingate 30 s	1-s peak power, 30 s mean power
van der Zwaard et al. [45]	CR	28 (28/0)	25 ± 7	Yes/No	VL	Volume	pCSA, FL, PA	Not reported	Wingate 30 s	Peak power during the Wingate at 100 Hz
Kordi et al. [47]	CR	35 (35/0)	22 ± 4	Yes/No	Quadriceps, hamstrings, VL	Volume	FL, PA	Linear extrapolation	Sprint cycling test	Peak power during the sprint cycling test
Coratella et al. [49]	CR	21 (21/0)	24 ± 4	Yes/No	VL, GM	N.A.	ACSA, FL, PA	Extended field of view	Wingate 30 s	Peak power during the Wingate
Lee et al. [50]	CR	24 (24/0)	20.7 ± 1	Yes/No	RF, VL, VI, VM, GM, GL	N.A.	MT, FL, PA	Trigonometric equation	Wingate 20 s	Mean power output
Lee et al. [42]	CR	58 (58/0)	20.1 ± 1.4	Yes/No	RF, VL, VM, TA, GM, GL	N.A.	MT, PA	N.A.	Wingate 30 s	Peak power during the Wingate
Cesanelli et al. [44]	CR	16 (16/0)	32.8 ± 8.2	Yes/No	RF, VL, VM	N.A.	N.A.	N.A.	Wingate 15 s	Peak power during the Wingate, 15 s mean power

Abbreviations: CR = cross-over trial; M = male; F = female; VL = vastus lateralis; VM = vastus medialis; VI = vastus intermedius; RF = rectus femoris; GM = gastrocnemius medialis; GL = gastrocnemius lateralis; TA = tibialis anterior; N.A. = not applicable; MT = muscle thickness; FL = fascicle length; PA = pennation angle; pCSA = physiological cross-sectional area; ACSA = anatomical cross-sectional area. Age is presented as overall or groupwise mean ± standard deviation, or as age range, if not otherwise reported in the studies

Muscle volume was only assessed in four studies, of which two employed MRI [43, 47] and the other two employed 3D ultrasonography [45, 46]. Three studies determined the CSA using 3D [45, 46] or 2D ultrasonography [47]. Four studies reported MTs [41, 42, 48, 50], six PAs [41, 42, 45–47, 49], and five FLs [45–47, 49, 50]. Only one article reported the echo intensity [44]. Regarding FL calculation, just three of the five studies indicated the procedures applied for its estimation. One study used the linear extrapolation method [47], another the extended field of view procedure [49], and the other a trigonometric equation [50].

### Sprint Performance Outcomes

Sprint performance was evaluated with the Wingate test in all the studies except for one [47] (Table 1). Most studies employed the classical 30-s Wingate test, while two used 15-s [44] and 20-s [50] all-out sprints. All the studies evaluated the peak power output, but only six assessed the mean power output [41, 43, 44, 46, 48]. Peak power output measurement varied among studies, i.e., peak (highest 1-s averaged) power output, maximum anaerobic power over 5-s, or peak power during the test. Only two studies reported the performance outcomes normalised by body weight [42, 49]. No study normalised the power output to the lean mass of the lower extremities.

### Associations between Muscle Morphology, Architecture, and Quality and Sprint Performance

The following associations shown here are only displayed if there were*n* ≥ 2 studies with common variables. Moderate (*r* = 0.59) to very strong (0.83) significant correlations were found between the muscle volume of the vastus lateralis with peak power output in absolute values [45, 46]. The CSA of the vastus lateralis showed moderate significant associations (*r* = 0.41 and 0.45) with peak power output in absolute values [45, 46].

Heterogeneous results regarding muscle architecture were found among the analysed studies. In this regard, a weak (*r* = 0.26) to very strong (*r* = 0.81) positive significant correlation was found between PA of the vastus lateralis with absolute peak power output [45, 47]. In contrast, two studies found non-significant associations (*r*= -0.02 to -0.015) between the PA of the vastus lateralis with absolute peak power output [42, 45]. Very weak negative non-significant (*r* = -0.15) [47] to a positive weak (*r* = 0.27) [42] and moderate (*r* = 0.60) [45] significant associations were reported between the FL of the vastus lateralis and absolute peak power output.

A weak (*r* = 0.32) to very strong significant correlation (Spearman´s rho = 0.87) was found between the MT of the rectus femoris and peak power output [42, 48]. Similar results were reported for MT of the vastus lateralis and peak power output (*r* = 0.162 to Spearman´s rho = 0.90) [42, 48]. Moderate (*r* = 0.58 and 0.59) to very strong (Spearman´s rho = 0.91) significant associations were observed for MT of the rectus femoris and mean power output [42, 48, 50]. Similarly, moderate (*r* = 0.37 and 0.48) to very strong (Spearman´s rho = 0.90) significant associations were shown for MT of the vastus lateralis and mean power output [42, 48, 50].

Lastly, one study reported associations between echo intensity and sprint performance variables [44]. This article found moderate negative significant associations between echo intensity and peak power output ranging from *r* = -0.54 to *r* = -0.62 depending on the evaluated muscle, i.e., vastus lateralis, rectus femoris, and vastus medialis.

Given that a subset of studies included regression analysis investigating the relationship between muscle architecture and cycling performance, a post hoc examination of these findings was conducted despite not being stipulated originally within the eligibility criteria. Only regressions featuring a minimum of two regressors were addressed based on the recognition that previous results have primarily depicted raw associations between two variables, whereas the inclusion of regressions allows for the presentation of adjusted associations.

In terms of the relationship between muscle volume and power output, one of the studies showed a significant association, indicating that vastus lateralis muscle volume largely accounted for peak power output (R^2^ = 0.82) in 12 males and 6 male and female Olympic rowers analysed conjointly [46]. The same authors reported a regression model to predict lean body mass (LBM)-normalised sprint performance, in which LBM was allometrically corrected by raising its value to the 2/3 exponent, i.e., sprint performance was expressed as W/kg LBM^2/3^ [45]. In the latter study, fast fibre-type percentage alongside vastus lateralis muscle volume explained 65% of the variance in normalised sprint performance [45]. Building on this, it has been shown that both quadriceps volume (76%) and vastus lateralis PA (11%) collectively explained 87% of the variance in peak power output in cyclists [47]. In agreement with the previous findings, it has been reported that the anatomical cross-sectional area of the vastus lateralis and gastrocnemius medialis accounted for 85% of peak power (normalised per body mass) variance in 21 amateur cyclists with large differences in muscle cross-sectional areas [49].

Using stepwise multiple regression, a close linear relationship between vastus lateralis, erector spinae, and rectus femoris MT and 5-s peak power output (absolute values) has been reported (R^2^ = 0.993, adjusted R^2^ = 0.989) in 12 cyclists with large differences in power output and muscle thickness [48]. Additional research using multiple regression analyses has reported that the MT of vastus lateralis and gastrocnemius medialis emerged as significant predictors of peak power in absolute values [42]. In the same study, rectus femoris MT (50% region) and gastrocnemius medialis MT were significant predictors of mean power in absolute values. Nevertheless, the authors did not provide information regarding the coefficients of determination or the percentage of variance explained by each muscle in their multiple regression analyses.

Furthermore, regarding FL, only one study identified the FL of the rectus femoris as a significant predictor of 20-s mean cycling power (absolute values) in varsity cyclists [50].

### Methodological Quality

**Fig. 2:**
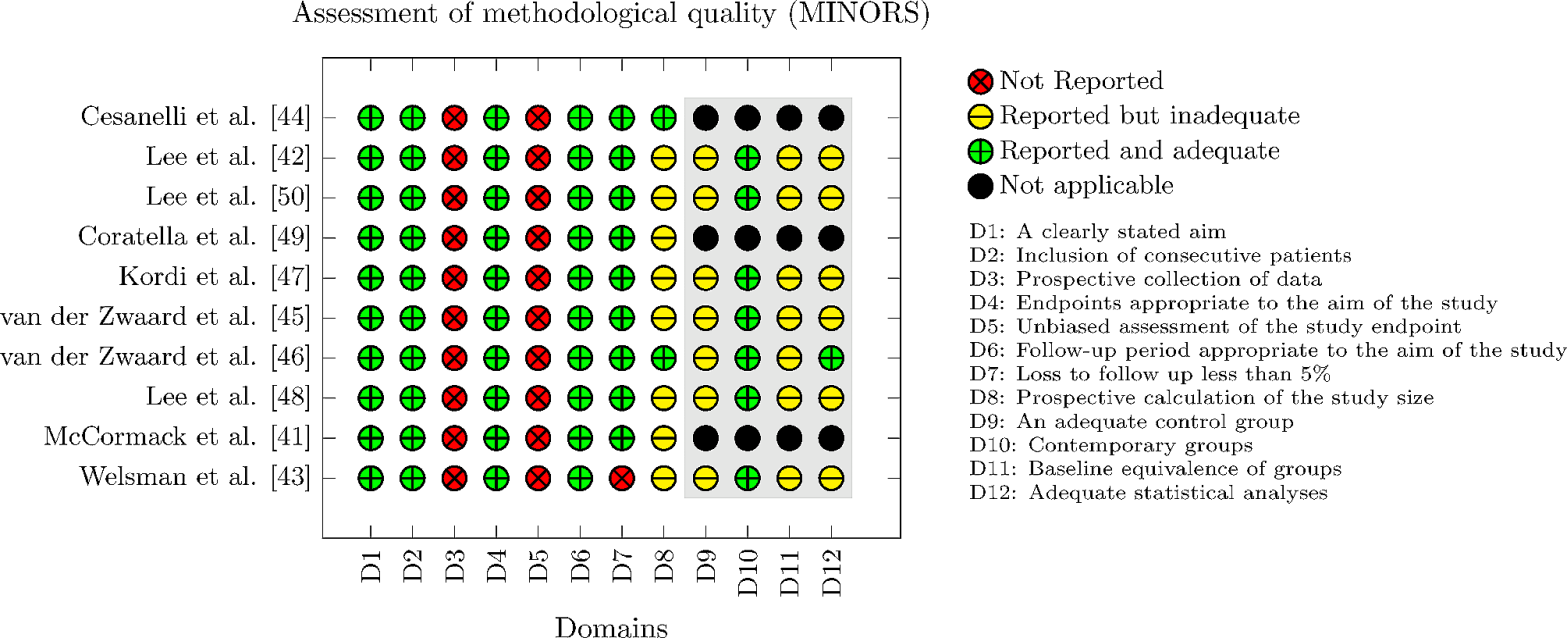
Methodological Index For Non-Randomized Studies (MINORS) plot. Each item is scored on a scale of 0 (red = not reported), 1 (yellow = reported but inadequate), and 2 (green = reported and adequate), with a higher score indicating better methodological quality. Black dot = not applicable

The quality of the studies was scored based on the MINORS scale designed for non-randomised studies (Fig. 2). Only three studies were classified as non-comparative studies, meaning it was not possible in these articles to evaluate the last four items. The mean score of the comparative studies (*n* = 7) was 16/24 (range: 14 to 18). The mean score of the non-comparative studies (*n* = 3) was 11.3/16 (range: 11 to 12). All studies scored 2 (i.e., reported and adequate) on items 1, 2, 4, 6, and 7. In addition, items 3 and 5 relating to the prospective data collection and unbiased assessment of the study endpoint scored 0 (i.e., not reported). Lastly, item 8 and the last four items related to additional criteria in the case of comparative studies were mostly scored with 1 (i.e., reported but inadequate).

## Discussion

This systematic review revealed that muscle morphology, architecture, and quality probably contribute to sprint cycling performance, but the nature of these relationships appears to be multifaceted and somewhat varied across studies. More specifically, our findings underscored the importance of muscle volume, as indicated by strong correlations with peak power output, emphasising the role of muscle size in force production and, therefore, cycling performance. The muscle architecture elements, including muscle thickness, pennation angle, and fascicle length, showed mixed associations with performance outcomes, reflecting the complex interplay of muscle architecture parameters in an applied sports setting. Finally, preliminary evidence on muscle quality, as assessed by echo intensity, suggested a potential negative association with performance, meaning that a lower echo intensity is associated with better performance. However, this domain is relatively underexplored (i.e., evidence from only one study) and warrants further investigation. The diversity of methodologies across the included studies, from diagnostic imaging techniques to sprint performance assessment protocols, added complexity to the interpretation of the findings.

### Muscle Morphology

The critical role of muscle morphology, particularly muscle volume and CSA, in physical performance has been underscored in a plethora of studies. The findings from this review are consistent with this established knowledge, showing a substantial correlation between these morphological parameters of the vastus lateralis and peak power output in sprint cycling. However, it should be noted that these associations are limited to a small number of articles that evaluated these associations [43, 45–47].

The correlation between muscle volume and peak power output aligns well with prior research. For instance, Fukunaga et al. [51] have highlighted the relationship between muscle volume and strength, demonstrating that larger muscles housing a higher number of muscle fibres have a greater capacity for generating force. This principle is particularly relevant in sprint cycling, which demands high-intensity, explosive force generation for success [1]. Additionally, previous research has shown that the larger the muscle volume, the higher the proportion of fast-twitch muscle fibres, which are responsible for explosive movements, as required in sprint cycling [52]. In addition, it has been observed that resistance training could lead to hypertrophy of these fast-twitch fibres [53], thereby increasing muscle volume and potentially enhancing peak power output in activities like sprint cycling. However, it is crucial to note that muscle volume and CSA do not exist in isolation and are influenced by numerous factors, such as training status, age, sex, and nutrition [51, 54]. For instance, muscle hypertrophy resulting from resistance training can increase muscle volume and CSA, contributing to enhanced performance in athletes [55]. It is essential to distinguish between physiological (pCSA) and anatomical (ACSA) cross-sectional areas since an increase in PA is associated with a larger increase in pCSA than ACSA [56]. While the pCSA was determined in two studies [45, 46], only one reported the ACSA [49], and most associations reported in the literature between muscle strength and CSA have used ACSA. Finally, some studies included multiple regression analyses showing a predominant role of quadriceps muscle volume, especially the vastus lateralis, as indicated by the high R^2^ of the relationship between quadriceps muscle volume (or vastus lateralis muscle volume) and peak power output (R^2^ from 0.65 to 0.82) [45–47]. However, it is essential to interpret these findings cautiously, considering variations in sample characteristics and measurement methods across studies. Additionally, other authors found that the anatomical cross-sectional areas of the vastus lateralis and gastrocnemius medialis accounted for 85% of peak power (normalised per body mass) variance [49]. However, the low number of regressors included in these articles could lead to a selection bias, as mentioned in the literature [57, 58].

While the findings of the studies included in this review are insightful, it is crucial to consider the methodological differences. For instance, the techniques used to measure muscle volume and CSA, such as 2D or 3D ultrasonography and MRI, can lead to substantial variation in measurement errors [59]. Unfortunately, these variations prevent a meaningful meta-analysis of the published studies. This underscores the urgent need for standardisation of measurement techniques in future research, a crucial step towards enhancing the reliability and comparability of findings in this field.

### Muscle Architecture and Quality

Key architectural features such as the PA and FL directly impact the force and velocity of muscle contractions, respectively, and could, therefore, potentially influence sprint cycling performance [16, 17]. This review found a weak to very strong positive correlation mainly between the PA of vastus lateralis and absolute peak power output. However, some studies reported very weak associations, indicating the presence of other influencing factors or variability in measurement techniques [17].

The association between PA and force production has been substantiated in the literature [56, 60]. Greater PAs are usually associated with a larger pCSA, potentially enhancing force production [16, 56]. A study by Aagaard et al. [56] demonstrated that resistance training can increase PA, potentially leading to greater force production. However, the relationship between PA and cycling performance may be influenced by other factors, such as the cyclist’s technique, fibre type distribution, and the type and intensity of training, which should be investigated in future research [61, 62]. The PA changes dramatically during muscle contraction and has been thought to influence muscle force generation [63]. However, Lieber challenges this long-held belief, arguing that PA is more of a packaging strategy, allowing short fibres to be packed into a confined volume. He suggests that, despite measurable changes in PA during muscle contraction, it may not have any significant functional impact. Therefore, the author recommends revising current biomechanical models to cease incorporating PA as a functionally significant factor. This recommendation is based on experimental studies showing considerable muscle fibre rotation during contraction, as well as studies showing a complex muscle-connective tissue composite structure [64]. However, this perspective might not apply to all muscles and simplifies the complex interplay within muscle mechanics.

Additionally, the results from this review indicate a strong relationship between MT of the vastus lateralis and rectus femoris with both peak power output and mean power output in sprint cycling. This is expected since MT is just a unidimensional indirect assessment of the CSA. In agreement, a close linear relationship between ultrasound-assessed MT and MRI-derived ACSA and volume before and after 12 weeks of resistance training (*r* = 0.82, *P* < 0.001 and *r* = 0.73, *P* < 0.001, respectively) has been reported [65]. These data support the use of MT as a surrogate of muscle ACSA. The robust association between MT and power output supports the use of resistance training to elicit muscle hypertrophy and enhance sprint cycling performance, as previously reported [66].

On the other hand, the present review shows varying results concerning the relationship between the FL of the vastus lateralis and absolute peak power output, ranging from very weak negative to moderate positive associations. This discrepancy may stem from the complex relationship between FL and cycling performance. Longer muscle fibres have more sarcomeres in series, which can produce faster contraction and benefit sprint cycling performance [15, 17]. However, the influence of FL on cycling performance may be confounded by several factors, including the cyclist’s specific pedalling technique and the distribution of muscle fibre types, which have been shown to influence cycling performance significantly [8].

Lastly, in this review, only one study assessed echo intensity in relation to sprint cycling performance [44]. This study found moderate negative associations between echo intensity and sprint performance variables, indicating that lower echo intensity (suggestive of higher muscle quality) might be associated with increased sprint performance. Previous research in older adults has shown an inverse relationship between echo intensity and physical performance, suggesting that higher levels of intramuscular fat and connective tissue (both of which increase echo intensity) might be detrimental to muscle function [23]. Similarly, in a study of young, healthy individuals, echo intensity was negatively associated with muscle strength and positively correlated with body fat percentage, indicating that lower muscle quality may be associated with reduced strength and higher adiposity [28]. Together, these findings point to a potentially relevant role of echo intensity in sprint cycling performance. However, given the limited research in this area, particularly in athletic populations, more studies are needed to explore this relationship further and determine the utility of echo intensity as a marker of muscle quality in athletes.

On the other hand, some of the included studies conducted multiple regression analyses, indicating that both MT and FL can predict peak and mean power output in sprint cycling, both in absolute and relative values, depending on the study [42, 48, 50]. In general, high regression coefficients were reported in studies with high heterogeneity in body sizes and performance levels in the analysed groups, mainly when power output was included in the regression models in absolute values. However, as with the muscle morphology variables, the low number of regressors used in these articles should be noted, which could lead to selection bias [57, 58]. Therefore, these findings should be interpreted cautiously, considering variations in sample characteristics and measurement methods across studies. Our eligibility criteria aimed to ensure consistency in data analysis methods, particularly regarding muscle architecture assessments.

These divergent findings underscore the need for further investigation using precise and standardised methodologies to assess FL. For instance, the linear extrapolation method, extended field of view, and the trigonometric equation have been used to calculate FL in the included studies, potentially influencing the variability in reported associations [67].

### Methodological Quality

The methodological quality of the included studies, as indicated by the MINORS scale, generally varied. All studies adequately reported the aim, endpoints, and inclusion of consecutive volunteers, indicating the rigorous design of these studies. However, prospective calculation of the study size and an unbiased assessment of the study endpoints were areas of limitation. The variation in imaging and sprint performance testing methods across studies adds complexity to comparing and interpreting the findings. Using standardised, reliable, and valid methods in future research would enhance the comparability of results across studies and improve the overall quality of the evidence [68].

### Limitations and Future Research

The present systematic review has uncovered notable heterogeneity across studies regarding several key methodological aspects. Differences in diagnostic imaging tools (MRI vs. ultrasound) [59], the lack of normalisation of power output to lean mass, muscle mass, or body weight [69, 70], the method employed for the estimation of fascicle length [67], or the utilisation of different performance tests impact the calculation of mean power output substantially (e.g., Wingate test or others), explain significant variation in the field. Given the scarcity of studies employing high-quality fascicle length measurements, their impact on sprint cycling performance remains elusive. Additionally, while it is well-documented that certain ergometers, such as the Lode Excalibur (Groningen, The Netherlands) and SRM (Jülich, Germany), are associated with low measurement errors, the variability in equipment across studies may introduce inconsistency in power and performance measurements. Since other ergometers exhibit higher measurement errors, this variability should be acknowledged as a limitation of interpreting and generalising our findings. This methodological divergence limits the ability to synthesise data effectively and perform meta-analyses. The heterogeneity underscores the necessity for adopting more standardised methodologies in future research to ensure comparability and validity of results. Future studies should consider all these limitations to enhance experimental designs and effectively address research questions.

### Practical Applications

The identified relationships between muscle morphology, architecture, and quality with sprint cycling performance offer valuable insights for athletes, coaches, and sports scientists. Understanding the importance of muscle volume, particularly its robust association with absolute peak power output, highlights the significance of muscle size in force production and, consequently, cycling performance. Coaches and athletes may benefit from this knowledge by incorporating targeted resistance training programs to increase muscle volume, especially in muscles like the vastus lateralis, to enhance sprint cycling performance. Additionally, insights into muscle architecture parameters such as muscle thickness, pennation angle, and fascicle length could inform the development of more specific training interventions tailored to individual athlete characteristics. For instance, athletes with lower muscle thickness or suboptimal pennation angles may benefit from targeted strength training exercises to improve these architectural features, and some studies indicate that eccentric strength training and stretching may increase fascicle length in specific muscles [71, 72]. However, it is essential to note that further research is needed to validate these practical applications and establish specific guidelines for implementation in athletic settings.

## Conclusions

This systematic review identified associations between muscle morphology, architecture, and sprint cycling performance, as evidenced by studies utilising varying methodologies. Key findings underscored: (1) the significant role of muscle volume and cross-sectional area in peak power output, while the influence of muscle architectural features appeared more complex; (2) while preliminary evidence suggests a potential association between echo intensity and sprint cycling performance, caution is warranted until this finding is replicated in the literature. Further investigation is needed to elucidate the role of echo intensity as a performance determinant in sprint cycling; (3) the heterogeneity among studies underlines a pressing need for standardisation in measurement techniques and performance tests to enhance comparability and enable robust meta-analyses, thereby providing clearer direction for future investigations and practical applications; (4) only 20% of the included studies normalised sprint performance outcomes by body weight, and none by the muscle mass of the lower extremities.

Given the reliance on simple analyses such as Pearson-product and Spearman correlations, the intricate nature of these relationships may not be fully captured. Advanced statistical techniques like mediation analysis may offer a more comprehensive understanding of the complex interplay between muscle characteristics and sprint cycling performance. In the end, intervention studies modifying the muscle mass and the rest of the architectural variables would be required to establish a link between changes in muscle phenotype and sprint performance. However, given the complexity of the variables that may influence sprint performance, including muscle phenotype [73] and neuromuscular factors [74], future research should adopt a more integrative approach.

### Electronic Supplementary Material

Below is the link to the electronic supplementary material.

Supplementary Material 1: PRISMA checklist

Supplementary Material 2: Search strings for electronic databases

Supplementary Material 3: Reports excluded for eligibility

## Data Availability

The data that support the findings of this study (data collection forms; data extracted from included studies; data used for all analyses; and any other materials) are available from the corresponding author upon reasonable request.

## Abbreviations

ACSA: Anatomical cross-sectional area
CSA: Cross-sectional area
FL: Fascicle length
LBM: Lean body mass
MINORS: Methodological Index For Non-Randomized Studies
MRI: Magnetic resonance imaging
MT: Muscle thickness
PA: Pennation angle
pCSA: Physiological cross-sectional area
PRISMA: Reporting Items for Systematic Reviews and Meta-analysis
