# Supplementary material for: Interplay of Muscle Architecture, Morphology, and Quality in Influencing Human Sprint Cycling Performance: A Systematic Review
Source: Sports Med Open. 2024 Jul 19;10:81. doi: 10.1186/s40798-024-00752-2 (PMC11258115; doi:10.1186/s40798-024-00752-2)
Supplement: Supplementary file 2 — Supplementary Material 2: Search strings for electronic databases [file 40798_2024_752_MOESM2_ESM.docx]

**Supplementary File II.** Search strings for electronic databases.

The original search string was created in PubMed. The search strings employed for SPORTDiscus, Web of Science, and CINAHL Complete were translated using an automatic online tool ([www.sr-accelerator.com/Polyglot](http://www.sr-accelerator.com/Polyglot)):

1. **Search strategy for PubMed**

URL: <https://pubmed.ncbi.nlm.nih.gov>

Filters: no filters were applied.

("sprint cycling"[All Fields] OR "sprint"[All Fields] OR "wingate"[All Fields] OR "anaerobic power"[All Fields]) AND ("Muscle architecture"[All Fields] OR "muscle thickness"[All Fields] OR "pennation angle"[All Fields] OR "fascicle length"[All Fields] OR "cross-sectional area"[All Fields] OR "echo intensity"[All Fields] OR "muscle volume"[All Fields] OR "ultrasound"[All Fields] OR "sonography"[All Fields] OR "magnetic resonance"[All Fields])

1. **Search strategy for SPORTDiscus (via EBSCOhost)**

URL: [https://web.s.ebscohost.com/ehost/search/advanced](https://web.s.ebscohost.com/ehost/search/advanced?vid=1&sid=bd37a48f-8a77-49a1-9374-b0c6f3945c63%40redis)

Filters: only academic journals

(TX "sprint cycling" OR TX "sprint" OR TX "wingate" OR TX "anaerobic power") AND (TX "Muscle architecture" OR TX "muscle thickness" OR TX "pennation angle" OR TX "fascicle length" OR TX "cross-sectional area" OR TX "echo intensity" OR TX "muscle volume" OR TX "ultrasound" OR TX "sonography" OR TX "magnetic resonance")

1. **Search strategy for Web of Science**

URL: <https://www.webofscience.com/wos/alldb/basic-search>

Filters: no filters were applied.

("sprint cycling" OR sprint OR wingate OR "anaerobic power") AND ("Muscle architecture" OR "muscle thickness" OR "pennation angle" OR "fascicle length" OR "cross-sectional area" OR "echo intensity" OR "muscle volume" OR ultrasound OR sonography OR "magnetic resonance")

1. **Search strategy for CINAHL Complete**

URL: <https://web.s.ebscohost.com/ehost>

Filters: no filters were applied.

("sprint cycling" OR sprint OR wingate OR "anaerobic power") AND ("Muscle architecture" OR "muscle thickness" OR "pennation angle" OR "fascicle length" OR "cross-sectional area" OR "echo intensity" OR "muscle volume" OR ultrasound OR sonography OR "magnetic resonance")
