# Supplementary material for: Interplay of Muscle Architecture, Morphology, and Quality in Influencing Human Sprint Cycling Performance: A Systematic Review
Source: Sports Med Open. 2024 Jul 19;10:81. doi: 10.1186/s40798-024-00752-2 (PMC11258115; doi:10.1186/s40798-024-00752-2)
Supplement: Supplementary file 3 — Supplementary Material 3: Reports excluded for eligibility [file 40798_2024_752_MOESM3_ESM.docx]

**Supplementary File III.** Reports excluded for eligibility.

**Reason 1:** Studies not investigating associations, i.e., using the Pearson product-moment correlation coefficient or Spearman´s rank correlation coefficient, between muscle architecture, morphology or quality, and sprint cycling performance.

1. Lin YC, Pandy MG. Predictive Simulations of Human Sprinting: Effects of Muscle-Tendon Properties on Sprint Performance. Med Sci Sports Exerc. 2022;54(11):1961-1972. <https://doi.org/10.1249/mss.0000000000002978>.
2. Kuki S, Konishi Y, Okudaira M, Takezawa K, Wakayoshi K. Sex differences in motor performance and anaerobic peak power of Japanese primary school children aged 11 to 12 years. J Hum Sport Exerc. 2022;17(3):567-575. <https://doi.org/10.14198/jhse.2022.173.08>.
3. Ando R, Tanji F, Ohnuma H, Ikeda T, Yamanaka R, Suzuki Y. Thigh Muscularity and Sprinting Performance of National‐Level Long‐Distance Runners. J Hum Kinet. 2022;81(1):65-72.
4. Bellinger P, Bourne MN, Duhig S, Lievens E, Kennedy B, Martin A, Minahan C. Relationships between Lower Limb Muscle Characteristics and Force-Velocity Profiles Derived during Sprinting and Jumping. Med Sci Sports Exerc. 2021;53(7):1400-1411.
5. Miller R, Balshaw TG, Massey GJ, Maeo S, Lanza MB, Johnston M, Folland J. The muscle morphology of elite sprint running. Med Sci Sports Exerc. 2021;53(4):804-815. <https://doi.org/10.1249/MSS.0000000000002522>.
6. Lievens E, Bellinger P, Van Vossel K, Vancompernolle J, Bex T, Minahan C, Derave W. Muscle typology of world-class cyclists across various disciplines and events. Med Sci Sports Exerc. 2021;53(4):816-824.
7. Ritsche P, Bernhard T, Roth R, Lichtenstein E, Keller M, Zingg S, Faude O. M. Biceps femoris long head architecture and sprint ability in youth soccer players. Int J Sports Physiol Perform. 2021;16(11):1616-1624.
8. Miyake Y, Suga T, Terada M, Tanaka T, Ueno H, Kusagawa Y, Isaka T. No Correlation Between Plantar Flexor Muscle Volume and Sprint Performance in Sprinters. Front Sports Act Living. 2021;3:671248. [https://doi.org/10.3389/fspor.2021.671248. eCollection 2021](https://doi.org/10.3389/fspor.2021.671248.%20eCollection%202021).
9. Magal M, Liette NC, Crowley SK, Hoffman JR, Thomas KS. Sex-Based Performance Responses to an Acute Sprint Interval Cycling Training Session in Collegiate Athletes. Res Q Exerc Sport. 2021;92(3):469-476.
10. Prinz B, Simon D, Tschan H, Nimmerichter A. Aerobic and anaerobic power distribution during cross-country mountain bike racing. Int J Sports Physiol Perform. 2021;16(11):1610-1615.
11. Bobbert MF, Casius LR, van der Zwaard S, Jaspers RT. Effect of vasti morphology on peak sprint cycling power of a human musculoskeletal simulation model. J Appl Physiol. 2020;128(2):445-455.
12. Bobbert MF, Casius LR, van der Zwaard S, Jaspers RT. Effect of vasti morphology on peak sprint cycling power of a human musculoskeletal simulation model. J Appl Physiol. 2020;128(2):445-455.
13. Zaras N, Stasinaki AN, Spiliopoulou P, Hadjicharalambous M, Terzis G. Lean body mass, muscle architecture, and performance in well-trained female weightlifters. Sports (Basel). 2020;8(5):67.
14. Nuell S, Illera-Domínguez V, Carmona G, Alomar X, Padullés JM, Lloret M, Cadefau JA. Sex differences in thigh muscle volumes, sprint performance and mechanical properties in national-level sprinters. PLoS One. 2019;14(11):e0224862.
15. Tottori N, Suga T, Miyake Y, Tsuchikane R, Otsuka M, Nagano A, Isaka T. Hip flexor and knee extensor muscularity are associated with sprint performance in sprint-trained preadolescent boys. Pediatr Exerc Sci. 2018;30(1):115-123.
16. Rice PE, van Werkhoven H, Merritt EK, McBride JM. Lower leg morphology and stretch-shortening cycle performance of dancers. J Appl Biomech. 2018;34(3):211-219.
17. Stock MS, Mota JA, Hernandez JM, Thompson BJ. Echo intensity and muscle thickness as predictors Of athleticism and isometric strength in middle‐school boys. Muscle Nerve. 2017;55(5):685-692.
18. Bex T, Iannaccone F, Stautemas J, Baguet A, De Beule M, Verhegghe B, Derave W. Discriminant musculo‐skeletal leg characteristics between sprint and endurance elite Caucasian runners. Scand J Med Sci Sports. 2017;27(3):275-281.
19. Handsfield GG, Knaus KR, Fiorentino NM, Meyer CH, Hart JM, Blemker SS. Adding muscle where you need it: non‐uniform hypertrophy patterns in elite sprinters. Scand J Med Sci Sports. 2017;27(10):1050-1060.
20. Stenroth L, Cronin NJ, Peltonen J, Korhonen MT, Sipilä S, Finni T. Triceps surae muscle-tendon properties in older endurance-and sprint-trained athletes. J Appl Physiol (1985). 2016;120(1):63-69.
21. Mangine GT, Fukuda DH, Townsend JR, Wells AJ, Gonzalez AM, Jajtner AR, Stout JR. Sprinting performance on the Woodway Curve 3.0 TM is related to muscle architecture. Eur J Sport Sci. 2015;15(7):606-614.
22. Mangine GT, Fukuda DH, LaMonica MB, Gonzalez AM, Wells AJ, Townsend JR, Hoffman JR. Influence of gender and muscle architecture asymmetry on jump and sprint performance. J Sports Sci Med. 2014;13(4):904.
23. Çakır Atabek H. Relationship between anaerobic power, vertical jump and aerobic performance in adolescent track and field athletes. J Phys Educ Sport. 2014;14(4):643-648.
24. Stafilidis S, Arampatzis A. Muscle–tendon unit mechanical and morphological properties and sprint performance. J Sports Sci. 2007;25(9):1035-1046.

**Reason 2:** articles must evaluate muscle morphology, architecture, and quality with the following diagnostic imaging devices: computed tomography, magnetic resonance imaging, or ultrasound.

1. Tounsi M, Aouichaoui C, Tabka Z, Trabelsi Y. Leg muscle volume and power development of Tunisian adolescents: Effect of socioeconomic status. Sci Sports. 2022;37(1):20-30.
2. Işildak K. Investigation the correlation of leg volume with anaerobic power and dynamic balance. Int J Appl Exerc Physiol. 2020;9(6):31-38.
3. Özkan A, Ersöz G, Köklü Y, Alemdaroğlu U, Kayihan G. The role of leg volume and leg mass in determining the anaerobic performance and isokinetic knee strength in male soccer players. Med Sport. 2015;68:193-207.

**Reason 3:** articles must not be reviews.

1. Douglas, J., Ross, A., & Martin, J. C. (2021). Maximal muscular power: lessons from sprint cycling. Sports Med Open, 7(1), 48. doi: 10.1186/s40798-021-00341-7.
